# The Treatment of Fractures from a Common-Sense Point of View

**Published:** 1907-11-30

**Authors:** 


### Points in Surgery.

### THE TREATMENT OF FRACTURES FROM A COMMON-SENSE POINT OF VIEW.

### XIV. FRACTURES IN THE NEIGHBOURHOOD OF THE KNEE-JOINT.

Almost every conceivable variety of fracture has been described as occurring in this situation. For all practical purposes these injuries may be classed in three groups, excluding fracture of the patella, which has already been dealt with. They are (1) supracondyloid fracture of the femur, (2) separation of the lower epiphysis of the femur, and (3) fractures involving the joint itself. The last class includes oblique fracture of one or other condyle; T-shaped fracture of the lower end of the femur, in which supracondyloid fracture is complicated by vertical separation of the two condyles; and oblique fracture of one or other tuberosity of the tibia, the line of which runs up into the joint. Separation of the upper epiphysis of the tibia is so rare an accident that it need not be considered.

Supracondyloid fracture of the femur resembles in its main characteristics fractures of the lower third of the shaft. For the displacement is the same, the lower fragment being flexed at the knee and dragged back behind the upper by the attachment of the two heads of the gastrocnemius muscle. The difference lies in the site of the lesion. In supracondyloid fracture it is situated roughly about two inches above the epiphysial line. It may be produced by indirect or by direct violence: the latter is most common, falling from a bicycle being quite a frequent cause. The propinquity of the line of fracture to the knee-joint complicates the treatment, because the lower fragment is often drawn upwards as well as backwards, and this necessitates the use of some form of extension. The most convenient form to employ is a Hodgen's splint, as advised for fractures of the lower third of the shaft; but it is more difficult to apply satisfactorily because the lower fragment is so small, and consequently tends to slip out of position again. As soon as union is fairly firm, generally in three or four weeks, the extension apparatus may be taken off and the limb put up on a Macintyre's splint. The great point in treatment is to start passive movement as early as possible. In fact, it may be laid down as a general rule that the nearer the site of a fracture is to a joint, the sooner should passive movement be started, if subsequent impairment of use of the joint is to be avoided. Wiring the fragments together is not to be recommended; the operation is difficult, and the results are not uniformly satisfactory. It should only be undertaken as a last resource after all other forms of treatment have been tried and failed. It may then be tried in cases where little or no union has taken place, and attempts to keep the fragments in position are completely unsuccessful. It is true that the operative treatment of simple fractures has been largely advocated in recent years. But the result has been that the method has been tried in all cases without discrimination, and the writer would once more lay stress on the fact that the treatment of injuries to bone by introducing what is, to all intents and purposes, a foreign body is unjustifiable in the absence of definite indications for its performance, or until all other methods have been tried and failed.

Separation of the lower epiphysis of the femur is a less common injury. It occurs from over-extension of the knee-joint in adolescents. It sometimes happens to small boys when they jump on the axle-bars of four-wheeled cabs. They hold on to the axle with their hands while they swing their feet forward under the cab. As the cab goes forward their leg is caught and hyperextended, and the shaft of the femur gives way at what is in them the weakest point, namely the epiphysial line. The results produced are quite characteristic. The lower epiphysis is cup-shaped, and when it is detached it is carried forwards on the femur, so that the lower end of the shaft is displaced backwards into the popliteal space. In severe cases it may press on, or even damage, the popliteal artery. As a general rule, when the epiphysis of a bone is detached the shaft is displaced in the direction of the main vessel of the limb at that point; and this case is no exception. It will be seen, therefore, that the displacement is exactly opposite to that met with in supracondyloid fracture. This fact, combined with the age of the patient, should make the diagnosis obvious. Satisfactory treatment is difficult, because the subsequent growth of the bone will be impaired, unless accurate coaptation of the fragments is obtained. This is best done without the aid of splints by flexing the knee under an anæsthetic to an angle of about 60°, keeping the limb in this position by means of a bandage round the thigh and ankle.

In the third class of case, in which the line of fracture involves the articular surface of the femur or the tibia, it is impossible, as a matter of practical experience, to diagnose the exact condition of the injury without a radiograph. Nor is it really very important to do so, as long as the presence of some such fracture is recognised. The injury is in nearly all cases associated with synovitis, and the limb is therefore kept somewhat flexed. The patient cannot bear complete extension, and it is necessary, therefore, to treat the fracture on an angular splint. The best splint to employ is a Macintyre, which consists of two metal troughs padded and movable at the knee by means of a hinge. It has also a movable footpiece. It should extend well up to the gluteal fold. The limb is laid on the splint, which is fixed at a convenient angle, and as soon as the effusion in the joint has subsided, which usually happens in a few days with evaporating lotion, although it may occasionally be necessary to aspirate the joint, passive movement must be begun. The important point is to try to obtain a useful limb for the patient, and to avoid a stiff knee. But after this injury it rarely happens that the injured limb is subsequently as good as the other one.

